# Fabrication of Functionalized Graphene Oxide–Aluminum Hypophosphite Nanohybrids for Enhanced Fire Safety Performance in Polystyrene

**DOI:** 10.3390/polym16213083

**Published:** 2024-10-31

**Authors:** Zhenzhen Deng, Tao Tang, Junjie Huo, Hui He, Kang Dai

**Affiliations:** 1 School of Environmental Science and Engineering, Guangdong University of Technology, Guangzhou 510006, China; 2 JIAHUA Special Cement Co., Ltd., Leshan 614003, China

**Keywords:** polystyrene, FGO–AHP nanohybrids, thermal property, fire safety performance

## Abstract

To enhance the fire safety performance in polystyrene (PS), a novel organic–inorganic hybrid material (FGO–AHP) was successfully prepared by the combination of functionalized graphene oxide (FGO) and aluminum hypophosphite (AHP) via a chemical deposition method. The resulting FGO–AHP nanohybrids were incorporated into PS via a masterbatch-melt blending to produce PS/FGO–AHP nanocomposites. Scanning electron microscope images confirm the homogeneous dispersion and exfoliation state of FGO–AHP in the PS matrix. Incorporating FGO–AHP significantly improves the thermal behavior and fire safety performance of PS. By incorporating 5 wt% FGO–AHP, the maximum mass loss rate (MMLR) in air, total heat release (THR), and maximum smoke density value (D_smax_) of PS nanocomposite achieve a reduction of 53.1%, 23.4%, and 50.9%, respectively, as compared to the pure PS. In addition, thermogravimetry–Fourier transform infrared (TG–FTIR) results indicate that introducing FGO–AHP notably inhibits the evolution of volatile products from PS decomposition. Further, scanning electron microscopy (SEM), FTIR, and Raman spectroscopy were employed to investigate the char residue of PS nanocomposite samples, elaborating the flame-retardant mechanism in PS/FGO–AHP nanocomposites.

## 1. Introduction

As a two-dimensional, one-atom-thick carbon material, graphene has attracted increasing attention in many fields due to its unique structure and mechanical and electrical properties [1]. However, the intrinsically strong van der Waals force and high surface area between graphene nanosheets give rise to the stacking and agglomeration in graphene, limiting its application in preparing high-performance materials [2]. To solve this issue, there has been a growing exploration to modify graphene surfaces and immobilize, anchor, and embed nanoparticles or other materials on graphene in different arrangements [3,4,5]. Benefiting from the numerous reactive groups (e.g., epoxide, hydroxyl, carboxyl, carbonyl groups, etc.), graphene oxide (GO), the precursor of graphene, has the unique advantage of being introduced into polymers to improve the mechanical and electrical properties [6]. Compared with zero-dimensional nanoparticles or one-dimensional nanowires, GO with a larger specific surface area displays improved dispersion in the polymer matrix [7]. However, one drawback of GO is its unstable thermal-oxidative properties, and the oxygen moieties on the GO surface can be easily destroyed by heating or chemical reagents, restricting its further application in some fields [8,9].

In recent years, surface modification has been utilized to enhance the thermal stability of GO and optimize its performance in polymer matrix by grafting functional molecules onto the GO surface [10,11]. For instance, organic halogen-free compounds, such as nitrogen and phosphorus-based flame retardants, have been grafted onto GO and exhibited enhanced flame-retardant efficiency in polypropylene [12] and epoxy [13]. It is worthy of note that 9,10-dihydro-9-oxa-10-phosphaphenanthrene-10-oxide (DOPO), a phosphorus-containing flame retardant with low toxicity, can exert flame retardancy in both condensed and vapor phases, significantly enhancing the fire safety of polymers [14]. In addition to catalyzing stable char formation, DOPO can produce P· and PO· radicals to quench the chain radical reaction of combustion, thereby enhancing the fire safety performance of polymers [15,16]. For instance, DOPO-phosphonimide grafted graphene nanosheets were introduced into epoxy resin, achieving significantly enhanced flame retardancy [17]. A multi-structured synergistic composite was synthesized by grafting nitrogen-containing polymers and DOPO on the surface of GO nanosheets, which demonstrated much-reduced heat release capacity (HRC) and significantly improved char yield [18].

As an extensively utilized plastic, PS exhibits many intriguing merits, including prominent chemical and thermal resistance, the convenience of processing and molding, and low cost, and has been widely applied in industrial fields such as foams [19], thermal insulation [20], and architecture [21]. However, PS is highly flammable, and the severe dripping accompanying the released toxic smoke during combustion greatly hinders the applications of PS in industry fields. Consequently, developing effective strategies to conquer these limitations and enhance the fire safety of PS is imperative. Recent studies have demonstrated that the combination of inorganic nanomaterials, including carbon nitride [22], layered double hydroxides (LDH) [23], and metal-loaded graphene [24], with organic compounds to prepare organic–inorganic nanohybrids can significantly enhance the fire resistance of polymer materials. Aluminum hypophosphite (AHP), a recently developed inorganic component with high phosphorus content, has been utilized as an effective flame retardant in a variety of polymer materials, including polybutylene terephthalate (PBT), polyethylene terephthalate (PET), and polyamide 6 (PA6) [25,26,27]. Lou et al. developed AHP@HNTs via the combination of halloysite nanotubes (HNTs) with aluminum hypophosphite using a one-pot method [28], and the prepared AHP@HNTs achieve superior flame retardancy in epoxy resin (EP) with a low addition (3 wt%), as evidenced by reductions in heat and smoke release. However, AHP also encounters drawbacks, including poor compatibility with polymer matrix and fire risk. Particularly, impact or heating can lead to the decomposing of AHP and release phosphine, which is spontaneously flammable in air, burning with a luminous flame, and even forming explosive mixtures in extreme cases. To conquer these limitations, melamine cyanurate encapsulated AHP (MCA@AHP) in polyamide 6 (PA6) was reported [29]. After microencapsulation, MCA showed a protection layer that inhibited the degradation of AHP and postponed the generation of phosphine, displaying high thermal stability and excellent inflammability.

Recently, the combination of graphene nanomaterials and other compounds to prepare composites/hybrids has exhibited intriguing properties in application [30,31]. Based on the organic-inorganic hybrid technology, FGO was combined with AHP to obtain an ideal flame retardant (FGO–AHP) with excellent performance in fire safety. The prepared FGO is a covalently functionalized GO, which was prepared by grating DOPO, a phosphorus-containing flame retardant, to GO. DOPO-containing additives and AHP as flame retardants are known to suppress combustion in both the condensed and gas phases [17,18]. The functionalization of GO greatly ameliorates the self-agglomeration of nanosheets in polymer composites, and the π-π conjunction between the benzene structures of DOPO and PS enhances the interaction with the matrix [17]. Moreover, the co-deposition of AHP to FGO can not only improve the thermal stability of graphene nanosheets but also facilitate the homogeneous dispersion of AHP and greatly enlarge its contacting area with the polymer matrix, fully exerting the flame retardancy efficiency in combustion. It is the first example of utilizing the co-deposition of AHP to FGO and the first incorporation of FGO–AHP into PS with high performance in fire safety. Then, the microstructure and thermal and flame-retardant behaviors of the resultant PS/FGO–AHP nanocomposites were characterized and discussed.

## 2. Experimental Section

### 2.1. Materials

Graphite powder (purity > 99.95%) and paraformaldehyde (HCHO) were obtained from Aladdin Industrial Corp. (Shanghai, China). Dimethylbenzene, N, N-dimethylformamide (DMF), triethylamine (TEA), and styrene were purchased from Damao Chemical Reagent Factory Co., Ltd. (Tianjin, China). 9,10-Dihydro-9-oxa-10-phosphaphenanthrene-10-oxide (DOPO) was supplied by Shenzhen Jinlong Chemical Co., Ltd. (Shenzhen, China). Phosphorus oxychloride (POCl_3_, AR), sodium hypophosphite (NaH_2_PO_2_, AR), and aluminum sulfate (Al_2_(SO_4_)_3_·18H_2_O, AR) were purchased from Shanghai Macklin Biochemical Co., Ltd. (Shanghai, China). All other chemicals were purchased from Guangzhou Chemical Co., Ltd. (Guangzhou, China) and used as obtained. Both DMF and TEA were dried with 4 Å molecular sieves before use. Polystyrene (PS, 158 K) was obtained from BASF-YPC Co., Ltd. (Nanjing, China). Other chemical reagents were used as received without further purification.

### 2.2. Fabrication of FGO and FGO–AHP

**Scheme 1:**
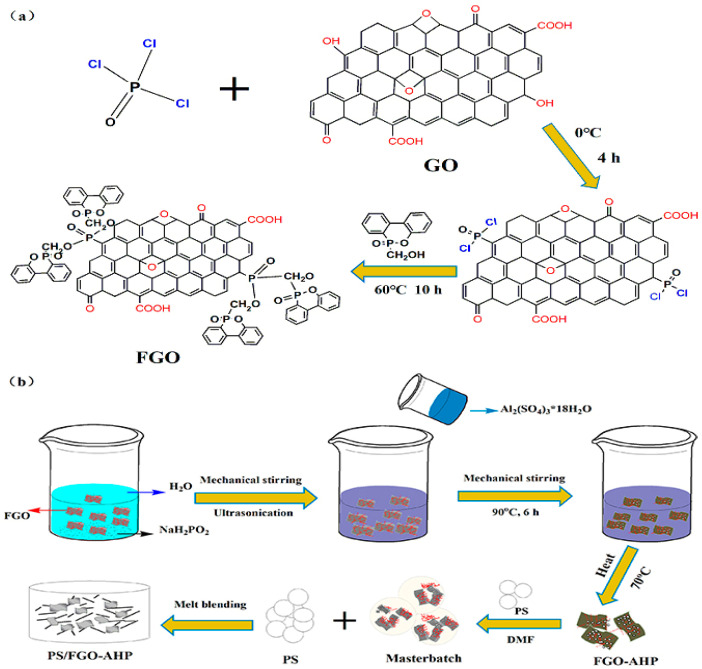
The fabrication process of (**a**) FGO, (**b**) FGO–AHP, and PS/FGO–AHP nanocomposites.

DOPO-OH was prepared from DOPO using a formaldehyde method [32]. The functionalized graphene oxide (FGO) was synthesized as follows: Briefly, POCl_3_ (0.06 mol, 9.252 g) was added into a three-necked round-bottom flask containing DMF (40 mL) with stirring under a nitrogen atmosphere at 0 °C. Triethylamine (0.2 mol, 20.258 g) dispersed in 10 mL DMF was dropped into the mixture with stirring. Then, 1.0 g of GO prepared from graphite was added to the flask, and vigorous mechanical stirring was maintained for 4 h, followed by setting and keeping at 60 °C for 20 min. Subsequently, 14.772 g of DOPO-OH dissolved in DMF (80 mL) was slowly dropped into the flask under vigorous stirring at 60 °C for 4 h. After that, the white powder DOPO-OH (0.06 mol, 14.772 g) was added to the flask. The mixture was kept vigorously stirred at 60 °C for 6 h with a nitrogen condition. After cooling, the crude product was filtered and washed 5 times with deionized water, followed by freeze-drying to obtain the product, coded as FGO (illustrated in Scheme 1a).

FGO–AHP was synthesized by a chemical deposition method as shown in Scheme 1b. In brief, 1.0 g of FGO and 7.922 g of NaH_2_PO_2_ were dispersed in 40 mL of deionized water by stirring and ultrasonication for 1 h. The system was conducted at 90 °C for 30 min. Then, 4.504 g of Al_2_(SO_4_)_3_·18H_2_O in 20 mL deionized water was added dropwise into the mixture with vigorous stirring. The reaction was allowed to continue at 90 °C for 6 h. Afterward, the resultant product was separated by filtration and rinsing copiously with deionized water. Finally, a black powder was obtained, which was then dried at 70 °C overnight, designated as FGO–AHP (illustrated in Scheme 1b).

### 2.3. Preparation of PS and PS Nanocomposites

PS nanocomposites with nanofillers (GO, FGO, and FGO–AHP) were prepared by masterbatch-melt blending (Scheme 1b). In a typical example, the preparation procedure of PS/FGO–AHP 2.0 was described as follows: PS spheres (9.0 g of PS and 1.0 g of FGO–AHP) were added to a three-necked flask equipped with DMF, following strong mechanical stirring and ultrasonication to obtain homogeneous suspension. FGO–AHP (1.0 g) was dispersed in 20 mL DMF by ultrasonication for 1 h and then was added to a three-necked flask equipped with a PS/DMF blend. After 2 h of ultrasonication and vigorous mechanical stirring, the obtained black slurry was dried under a vacuum at 120 °C for 12 h, cut into small granules, and further dried at 130 °C for 6 h.

Subsequently, all samples of the masterbatch were mixed with PS by melt blending at 180 °C at a constant speed of 50 rpm for about 8 min. PS/GO, PS/FGO, and PS/FGO–AHP were prepared on an XSS-300 twin-roller mill (SU-60ML, Shanghai Kechuang Rubber and Plastic Machinery Equipment Co., Ltd., Shanghai, China). The samples were hot-pressed into sheets with a thickness of 3.0 mm at 190 °C under 10 MPa for 10 min. According to the amount of GO/FGO/FGO–AHP in PS composites, the prepared samples are referred to hereafter as PS/GO2.0, PS/FGO2.0, and PS/FGO–AHP (2.0, 3.0, 4.0, and 5.0).

### 2.4. Characterization

The X-ray diffraction (XRD) patterns were recorded on a D/MAX-IIIC X-ray diffraction instrument using Cu Kα radiation (40 kV, 300 mA) from 5° to 80°. Fourier transform infrared (FTIR) spectra were recorded in the 4000–500 cm^−1^ region on a Nicolet 6700 spectrometer (Nicolet Instrument Company, Waltham, MA, USA) using the KBr discs method. The morphologies of the samples were examined via a SEM solver p47 pro using charge contrast imaging mode. Transmission electron microscopy (TEM) analysis was performed on a JEM-2100 transmission electron microscope at 200 kV. Thermogravimetric analysis (TGA) was conducted on a PE TGA-7 calorimeter from room temperature to 700 °C with a linear heating rate of 20 °C/min under nitrogen or air atmosphere, and all samples were kept with 5–10 mg. Micro combustion calorimeter (MCC) test was carried out in an MCC-2 (Govmark Inc., New York, NY, USA). According to ASTM D 7309, 5–7 mg of powdery samples were heated to 700 °C at a heating rate of 1 °C/s in an inert gas steam (80 mL/min). NBS smoke density chamber test (NBS Test) was conducted according to ISO 5659-2 standard, and the smoke density of the specimens was tested by an NBS smoke density test chamber (model SDB, Kunshan Modisco Combustion Technology Instrument Co., Ltd., Kunshan, China) with a heat flux of 25 kW/m^2^. Thermogravimetric analysis/infrared spectrometry (TG-IR) of PS and PS/FGO–AHP nanocomposite was performed using a TGA Q5000 IR thermogravimetric analyzer that was interfaced with a Nicolet IS 50 FTIR spectrophotometer. The sample was put in an alumina crucible and heated from room temperature to 800 °C at a heating rate of 20 °C/min under nitrogen. Raman spectroscopy measurements were conducted at room temperature using a Lab RAMHR 800UV Laser Raman spectrometer with a laser wavelength of 633 nm, and the Gaussian peak type in the Peak Fitting Module of Origin 8.0 software was employed in curve fitting to determine spectral parameters.

## 3. Result and Discussion

### 3.1. Characterization of GO, FGO, and FGO–AHP

**Figure 1:**
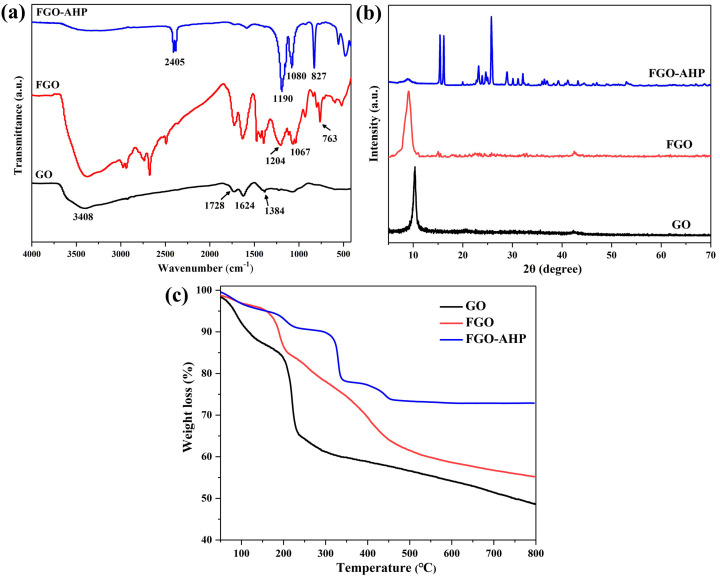
(**a**) FTIR and (**b**) XRD spectra of GO, FGO, and FGO–AHP and (**c**) TG curves of GO, FGO, and FGO–AHP in nitrogen.

The functionalized groups on GO, FGO, and FGO–AHP were investigated by FTIR spectroscopy measurements, as illustrated in Figure 1a. The spectrum of GO exhibits several characteristic absorption peaks, including the stretching vibration of O-H (3408 cm^−1^), C=O (1728 cm^−1^), C=C (1624 cm^−1^), C-OH (1384 cm^−1^), and C-O (1130 cm^−1^) [33]. In contrast, some new peaks can be seen in the spectrum of FGO. The peaks at 3000 cm^−1^–2500 cm^−1^ are attributed to C-H stretching vibration, and a new band appeared at 1204 cm^−1^, which can be ascribed to the P=O characteristic peak in DOPO [16]. In addition, the bands located at 1067 and 763 cm^−1^ are attributed to the stretching vibration of P-O-Ph in DOPO and the stretching vibration of aromatic rings, respectively [34]. The results demonstrate that DOPO-OH has been successfully grafted onto the surface of GO. For the FGO–AHP spectrum, the absorption peak of -OH at 3408 cm^−1^ decreases significantly, and the band of C-OH at 1384 cm^−1^ disappears, suggesting the hydrophobic nature of FGO–AHP. Furthermore, the FTIR spectrum of FGO–AHP reveals the characteristic peaks of AHP at 2405 (stretching vibration of -PH_2_), 1190 (bending vibration of -PH_2_), and 1080 cm^−1^ (P-O vibration) [35], indicating that AHP has been successfully loaded onto FGO.

Further, the structure of these samples was investigated using X-ray diffraction (XRD), and the corresponding XRD patterns are shown in Figure 1b. In the GO sample, a sharp peak at 2θ = 10.3° corresponding to the (002) graphitic lattice diffraction is observed, indicating an interlayer spacing of 0.796 nm [36]. Additionally, a weak peak at 42° is attributed to the (100) reflection in GO. In the FGO pattern, the (002) characteristic peak is shifted to 8.9°, suggesting an enlarged interlayer spacing due to the intercalation of functional phosphate and DOPO groups into the GO nanosheets. Furthermore, in the XRD of FGO–AHP, the characteristic (002) diffraction peak of FGO is much weakened, indicating significant deposition of AHP on the surface of FGO and hindering the penetration of X-ray into the interior of FGO–AHP [37]. In addition, the observed peaks between 15 and 55° can be attributed to the diffraction of AHP [38], further confirming the existence of AHP in FGO–AHP.

The thermal stability of GO, FGO, and FGO–AHP was characterized by TGA under a nitrogen atmosphere, and the corresponding TG curves are shown in Figure 1c and Table 1. A severe weight loss below 250 °C is seen for GO, giving rise to a 48.2 wt% char residue at 800 °C. This unstable thermal behavior is due to the existence of numerous oxygen-containing groups on the GO surface, which accelerate the degradation of GO in high temperatures [39]. In contrast, FGO exhibits significantly improved thermal behavior, as the T_0.1_ (the temperature at 10% weight loss) and T_0.2_ (the temperature at 20 wt% weight loss) are much higher than those in GO. This improvement can be attributed to the removal of oxygenous groups via reacting with the introduced functional groups. Notably, the hybridization of FGO and AHP contributes to the highest T_0.1_ and T_0.2_ in FGO–AHP, and the observed 73.6 wt% char residue corroborates the strong charring ability in FGO–AHP.

**Figure 2:**
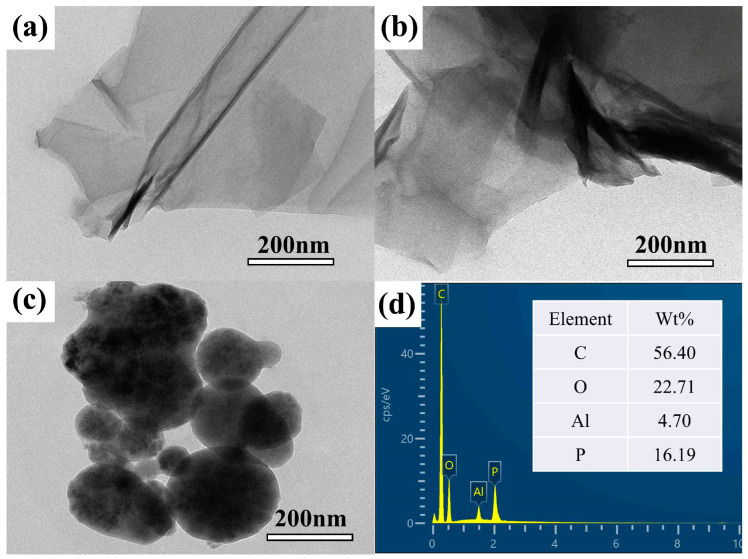
TEM images of (**a**) GO, (**b**) FGO, (**c**) FGO–AHP, and (**d**) EDS image of FGO–AHP.

TEM is a widely utilized technique for studying the structure of nanomaterials and their dispersion state within a polymer matrix. The TEM images of the structure of GO, FGO, and FGO–AHP are shown in Figure 2. It is clearly observed that GO exhibits a transparent smooth lamellar structure (Figure 2a), indicating the presence of large single GO sheets due to their extremely thin nature and the presence of oxygenous groups. For FGO, some black shadows are observed on the nanosheets (Figure 2b), suggesting that the functional phosphorus-containing molecules were grafted onto the surface, as confirmed by FTIR results. In Figure 2c, spherical morphology in FGO–AHP can be observed, with a sophisticated distribution in surface homogeneity [40]. The dark speckles observed in FGO–AHP are assumed to be AHP. This indicates that AHP molecules were deposited onto the FGO surface to form the FGO–AHP nanohybrids. Further, the element types and relative contents of FGO–AHP are tabulated as shown in Figure 2d, and the detected Al and P elements in this EDS image indicate the introduced phosphate groups and AHP in the material.

**Figure 3:**
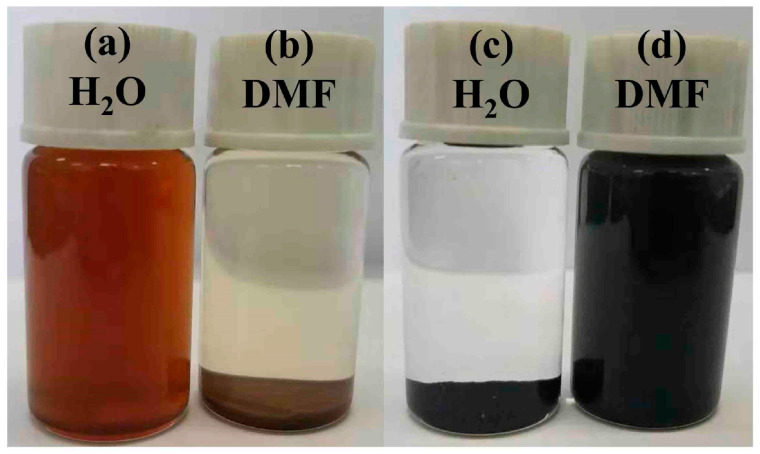
Optical pictures of (**a**,**b**) GO and (**c**,**d**) FGO–AHP in H_2_O and DMF after ultrasonication and two days of standing.

Furthermore, the dispersibility of GO and FGO–AHP in deionized water and DMF solvent was assessed. Photos of the dispersion states are shown in Figure 3. GO is readily exfoliated in aqueous solution due to the presence of numerous hydrophilic groups (e.g., carboxyl and hydroxyl groups), leading to the formation of stable colloidal suspensions of nanosheets [32]. After ultrasonication and two days of standing, the GO is stably dispersed in H_2_O but settles at the bottom of the DMF solvent, as shown in Figure 3a,b. On the other hand, FGO–AHP is well dispersed in the highly polar DMF solvent, forming a deep black solution. This phenomenon can be attributed to the strong polarity of AHP, which facilitates the dispersion of FGO–AHP in DMF.

### 3.2. Morphological Analysis

**Figure 4:**
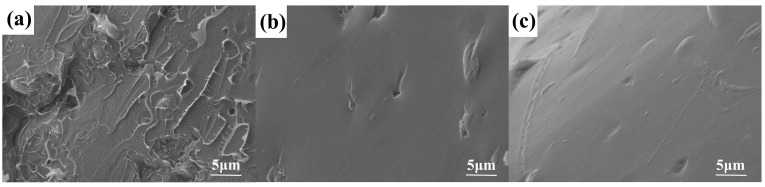
SEM images of the fractured surfaces of (**a**) PS/GO2.0, (**b**) PS/FGO2.0, and (**c**) PS/FGO–AHP2.0.

It is well established that the interfacial interaction between nanohybrids and polymer matrix plays a crucial role in influencing the properties of polymer nanocomposites [41,42]. The fracture surfaces of PS nanocomposite samples were characterized by SEM to analyze the dispersion of GO, FGO, and FGO–AHP in the PS matrix. As depicted in Figure 4a, the freeze-fractured surface of PS/GO2.0 is rough. The strong van der Waals attractions among GO nanosheets can account for this unfavorable interfacial interaction between the GO and PS matrix [43]. In contrast, the fracture surface of PS/FGO2.0 exhibits rarely embedded particles (Figure 4b), demonstrating the desirable compatibility between FGO and PS. This is likely due to the covalently functionalized molecules on the surface of FGO, which contribute to better dispersion in the PS matrix. Furthermore, the incorporation of FGO–AHP results in integrated nanohybrids within the PS matrix without aggregation or stacked graphene nanosheets (Figure 4c), indicating a strong interfacial interaction between the decorated AHP on the surface of nanohybrids and the PS matrix. This analysis confirms the favorable compatibility of FGO–AHP in the PS matrix, which is expected to enhance the thermal stability of PS/FGO–AHP nanocomposites.

### 3.3. Thermal Stability

**Figure 5:**
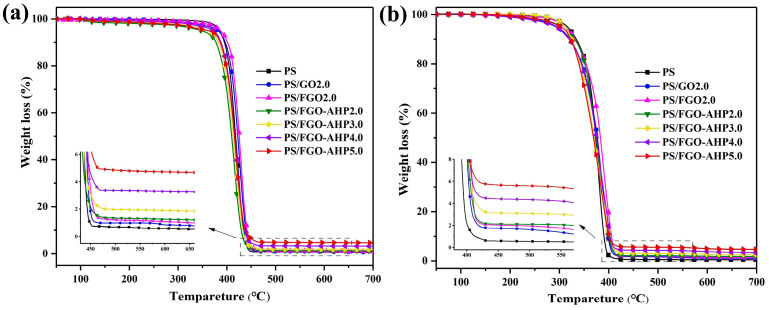
TG curves of neat PS and PS nanocomposites in (**a**) nitrogen and (**b**) air condition.

The TG curves and detailed data of neat PS and PS nanocomposites are presented in Figure 5 and Table 2, respectively. Under an N_2_ condition, the TG curves show that all samples undergo a one-stage thermal decomposition process in the temperature range of 320–470 °C, corresponding to the degradation of the chain-scissoring process in PS molecular chains [44]. The primary weight loss observed in neat PS is attributed to the release of CO, CO_2_, H_2_O, and hydrocarbon fragments [16], and similar decomposition behavior can be seen in PS/GO2.0 and PS/FGO2.0, suggesting the addition of GO and FGO has a limited effect on the initial thermal stability of PS, with a T_0.1_ of 397 and 401 °C for PS/GO2.0 and PS/FGO2.0, respectively. Notably, the 0.89 wt% char yield of PS/FGO2.0 at 700 °C is higher than that of PS/GO2.0 (0.61 wt%), indicating the improved thermal stability of FGO. With the incorporation of FGO–AHP, the decomposition behavior of PS/FGO–AHP nanocomposites is significantly altered. In Table 2, results indicate that the addition of FGO–AHP decreases the initial decomposition temperature (T_0.1_) (approximately 7–17 °C lower than that of pure PS). This phenomenon mainly arises from the decomposition of AHP at low temperatures to produce aluminum pyrophosphate and phosphine [35]. The generated aluminum pyrophosphate combined with the functionalized graphene nanosheets can act as a more effective physical barrier to prevent the PS matrix from degradation. Consequently, the nanocomposites containing FGO–AHP exhibit much-improved thermal stability at high temperatures, as the char residue of PS/FGO–AHP5.0 reaches 4.66 wt%, significantly higher than the limited 0.53 wt% char residue of neat PS. Moreover, compared with the neat PS, a slight reduction in the T_0.1_ value of all PS nanocomposites is observed in the air. However, the T_max_ (the temperature at the maximum mass loss rate) value of PS nanocomposites is increased with a lower maximum mass loss rate (MMLR), indicating that incorporating these nanomaterials inhibits the thermo-oxidation degradation of PS. Meanwhile, a higher char yield can be seen for all PS nanocomposites, compared to that in the nitrogen condition. For FGO–AHP nanohybrids, their homogeneous dispersion in PS facilitates the interaction between FGO–AHP and the matrix. After the decomposition of AHP, the generated phosphine is easily combined with the oxygen in the air to form phospho-acid, which acts as a flame retardant to catalyze the char formation of these nanocomposites rather than decomposition [35]. In addition, the graphene nanosheets not only act as a physical barrier to hinder the decomposition of the matrix but also provide adequate specific surface area for the reactions between phosphorous-containing compounds and PS molecules [45]. These effects contribute to a much-decreased MMLR and a notably enhanced char yield in PS/FGO–AHP nanocomposites, as presented in the TGA results (see Table 2).

### 3.4. Flammability of PS Nanocomposites

**Figure 6:**
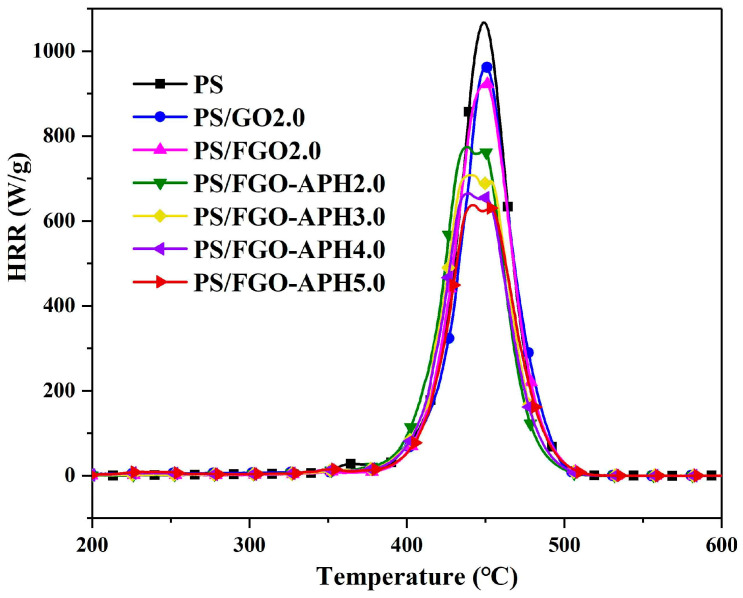
HRR curves of PS and PS nanocomposites.

The flammability of PS nanocomposites was characterized by Microscale Combustion Calorimetry (MCC), which is employed to measure the heat release, reflecting the combustion behavior of the materials, such as heat release rate (HRR), total heat release (THR), and heat release capacity (HRC), etc. The HRR curves of PS and PS nanocomposites and corresponding MCC data are shown in Figure 6 and Table 3. The neat PS presents a high peak of heat release rate (PHRR) value (1063 W/g), indicating its highly flammable nature. With the introduction of GO and FGO, the heat release of PS nanocomposites was retarded, as the PHRR values of PS/GO2.0 and PS/FGO2.0 are reduced to 964 and 925 W/g, respectively. Meanwhile, the PS/FGO–AHP nanocomposites demonstrate much decreased PHRR. Along with the increasing amount of FGO–AHP in PS, the PHRR values of PS/FGO–AHP nanocomposites (PS/FGO–AHP2.0, PS/FGO–AHP3.0, PS/FGO–AHP4.0, and PS/FGO–AHP5.0) exhibit a reduction of 27.2%, 33.4%, 37.5%, and 39.9%, respectively, as compared with that of virgin PS. Similarly, a significant decrease in THR can also be seen in PS/FGO–AHP nanocomposites. The THR value shifts from 45.6 kJ/g (PS) to 34.9 kJ/g (PS/FGO–AHP5.0), exhibiting a reduction of 21.5%. The following effects can account for the high efficiency of FGO–AHP in reducing heat release: FGO nanosheets act as a robust physical barrier during combustion, which catalyzes char formation and restrains the thermal degradation of the polymer matrix. Moreover, phosphate and pyrophosphate, the decomposition product of AHP, combine with FGO to form a solid physical shield, slowing down the heat and mass transfer between the gas and condensed phases and preventing further degradation of the underlying polymer matrix [46]. In addition, a significant reduction in HRC is observed, and the maximum reduction in HRC (PS/FGO–AHP5.0) reaches 40.3%. This achievement is attributed to the much-reduced MMLR and the combustion heat at the decomposition temperature [32]. As can be seen in Table 4, the flame retardancy of FGO–AHP is superior to that of the listed flame retardants, indicative of its high efficiency in PS nanocomposites.

To sum up, introducing FGO–AHP into PS significantly improves the flame-retardant properties of PS nanocomposites. Based on the MCC results, FGO and AHP demonstrate a favorable flame-retardant synergistic effect in PS.

### 3.5. Toxic Smoke and Gaseous Volatiles Analysis

**Figure 7:**
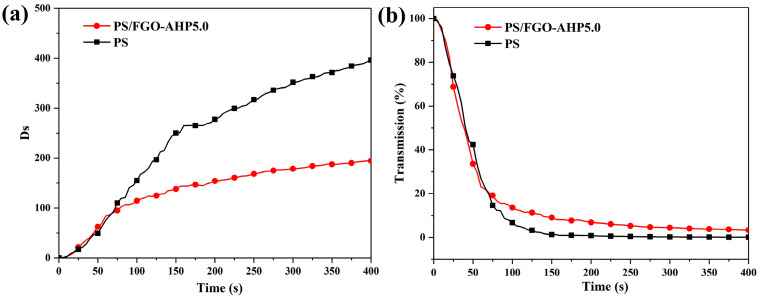
(**a**) Specific optical density and (**b**) light transmission curves for PS and PS/FGO–AHP5.0.

In the event of a fire, in addition to the heat release, the production of smoke, smoke particulates, and toxic gases during combustion is also life-threatening. To evaluate the smoke emission behavior of PS and PS nanocomposite, the NBS smoke density chamber test was conducted. The results, shown in Figure 7, indicate that the maximum smoke density value (Ds_max_) of pure PS is 396.3, whereas PS/FGO–AHP5.0 is significantly reduced to 194.6. This reduction is due to the combination of FGO and AHP, which lead to a decrease in smoke production. Higher transmittance values in the test indicate better visibility in the event of a fire. As shown in Figure 7b, the transmittance of pure PS is only 0.98% at 180 s (prime time for rescue in a fire), while the transmittance of PS/FGO–AHP5.0 is elevated to 7.9%. This suggests that the flame retardant FGO–AHP effectively reduces the smoke release of PS, providing more opportunities for escape during a fire.

**Figure 8:**
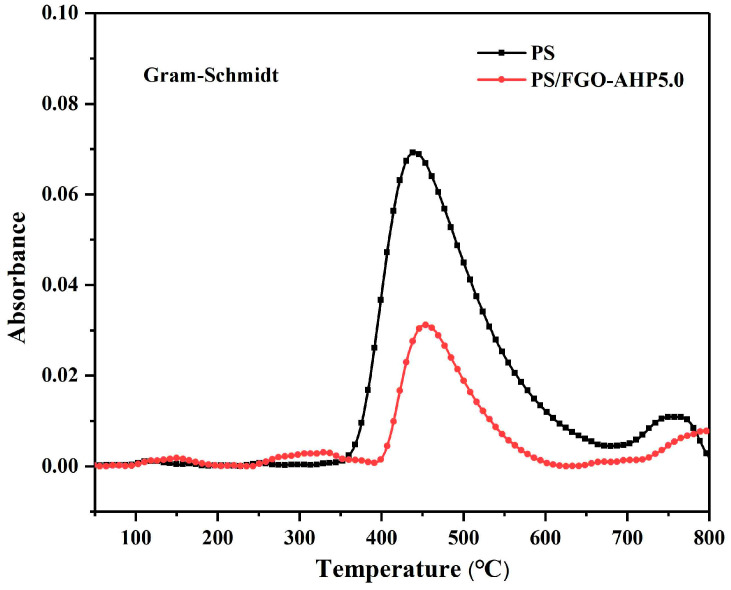
Gram–Schmidt (GS) curves of PS and PS/FGO–AHP5.0 nanocomposites.

Further, TG-IR was employed for continuous monitoring of gas variation with time and weight of residues, which is helpful for comprehension of the differences in thermal degradation mechanisms between pure polymers and their composites. Figure 8 illustrates the Gram–Schmidt (GS) curves for PS and PS/FGO–AHP5.0, and the results represent the evolution of the total pyrolysis gas products of the materials. It can be seen that the addition of FGO–AHP significantly reduces the total pyrolysis gas production of PS. In addition, the observed absorption peak at 335 °C for the PS/FGO–AHP5.0 nanocomposite corresponds to phosphine generated from the degradation of aluminum hypophosphite, which is consistent with the weight loss of PS/FGO–AHP5.0 in TG results.

Previous studies have demonstrated that under inert conditions, neat PS decomposes mainly into monomers, dimers, and trimers of phenyl alkenyl groups as aromatic compounds [50]. The absorbance versus temperature curves of the pyrolysis products of PS and PS/FGO–AHP5.0 are shown in Figure 8. A significant decrease in the absorbance intensity of combustible volatiles (hydrocarbons and aromatic compounds) is observed in PS/FGO–AHP5.0 compared to those of pure PS, indicating that the pyrolysis process of the PS molecular chain is inhibited. This improvement is assigned to the barrier effect of the well-dispersed FGO–AHP nanohybrids and the catalytic char formation. At high temperatures, aluminum pyrophosphate, the decomposition product of AHP, covers the surface of GO nanosheets, enhancing the physical shielding effect to protect the polymer matrix from degradation, and the produced phosphors-containing acid from AHP promotes the carbonization of decomposition products. Meanwhile, numerous PO ·and P· free radicals generated by DOPO and aluminum hypophosphite decomposition can capture H ·and OH· free radicals, quenching the combustion chain reaction [51]. As the combustible volatiles from PS pyrolysis diminish, the HRR and Ds values will decline. These results are in accordance with the MCC and smoke density test results.

**Figure 9:**
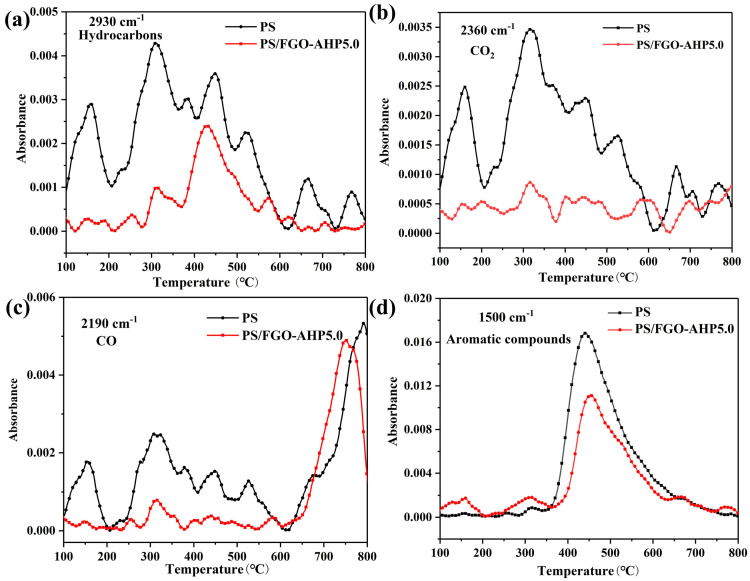
The absorbance of pyrolysis products vs. temperature for neat PS and PS/FGO–AHP5.0: (**a**) hydrocarbons, (**b**) CO_2_, (**c**) CO, and (**d**) aromatic compounds.

Furthermore, absorbance intensities of pyrolysis products for neat PS and PS/FGO–AHP5.0 vs. temperature are exhibited in Figure 9. It is observed that PS/FGO–AHP5.0 exhibits a significantly reduced absorption capacity for smoke and toxic gases (CO_2_ and CO), as compared to pure PS, indicating the excellent smoke suppression effects of FGO–AHP nanohybrids as well as the strong interfacial bonding with the material matrix. Therefore, the lower heat release value and excellent flue gas shielding efficiency confirm that PS/FGO–AHP is a reliable fire-retardant nanomaterial. It is also observed that PS/FGO–AHP5.0 exhibits significantly decreased hydrocarbon and aromatic compound intensities compared with those of pure PS, further corroborating the strong barrier effect of FGO–AHP nanohybrids.

### 3.6. Char Residue Analysis

**Figure 10:**
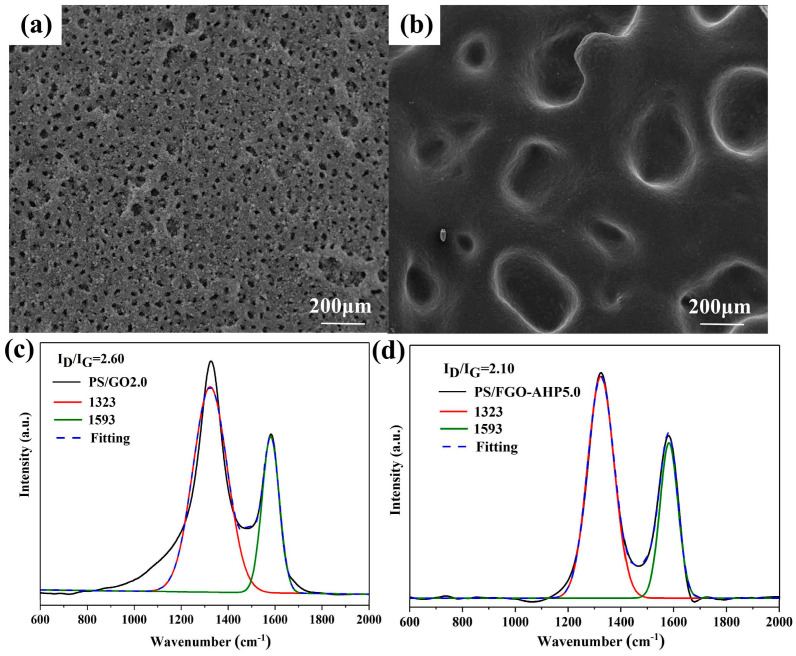
SEM images of char residues: (**a**) PS/GO2.0 and (**b**) PS/FGO–AHP5.0; Raman spectra of the residual char of (**c**) PS/GO2.0 and (**d**) PS/FGO–AHP5.0.

Figure 10a,b shows digital SEM images of the residual char for PS nanocomposites. It is clearly shown that the residual char of PS/GO2.0 appears as an irregular surface with numerous small holes and pits with a loose structure (Figure 10a), which can act as channels to facilitate the exchange of oxygen, heat, and combustible gas products between the burning matrix and the flame zone. As observed from Figure 10b, PS/FGO–AHP5.0 appears a continuous and smooth surface with a compact structure, suggesting a superior barrier effect during the polymer combustion. The above results confirm the favorable flame-retardant effect on PS-AHP nanocomposites.

Furthermore, the char residues of PS/GO2.0 and PS/FGO–AHP5.0 were obtained by heating the samples in a muffle furnace at 600 °C for 5 min, which were characterized by Raman spectroscopy. The spectra of PS/GO2.0 and PS/FGO–AHP5.0 were fitted into two peaks, corresponding to the D band (1323 cm^−1^) and the G band (1580 cm^−1^), respectively (Figure 10c,d). The G band represents the first-order scattering E_2g_ phonon of a sp^2^ hybrid carbon atom, whereas the D band is assigned to a breathing mode of k-point photons with A_1g_ symmetry, which accounts for the oscillation of the rocking carbon atom of disordered carbon or glassy carbon layer [52]. The integrated area ratio of the D band to the G band (I_D_/I_G_) can be employed to assess the degree of disorder and crystal size of graphitization in carbon materials. A lower I_D_/I_G_ value represents a better microstructure in the char residue with fewer defects. In Figure 10c,d, the I_D_/I_G_ ratios of PS/GO2.0 and PS/FGO–AHP5.0 are 2.60 and 2.10, respectively, suggesting a higher graphitization degree and higher thermally stable char structure for PS/FGO–AHP5.0. This improvement is attributed to the grafted flame-retardant molecules and anchored AHP molecules on the GO surface, which catalyze the formation of a cohesive char layer to protect the GO nanosheets from severe damage, resulting in an improved microstructure in char residue.

**Figure 11:**
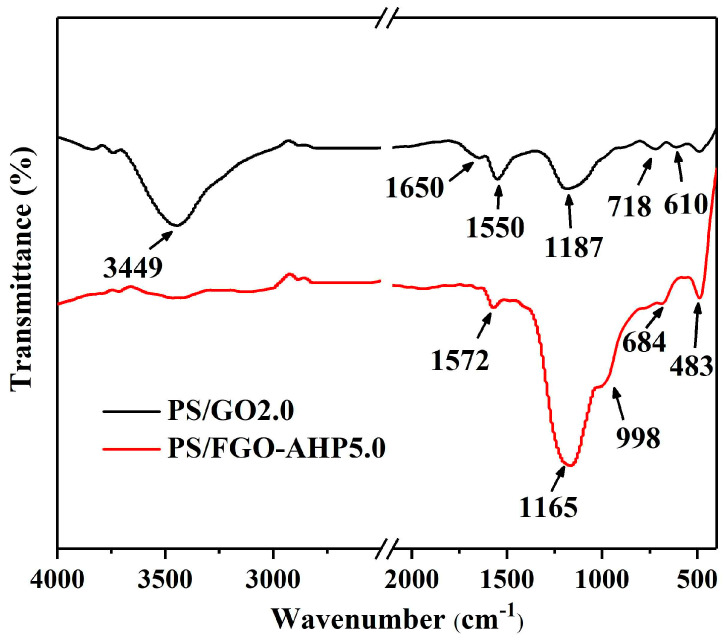
FTIR spectra of char residues for PS/GO2.0 and PS/FGO–AHP5.0.

To further understand the flame-retardant action of PS/FGO–AHP nanocomposites in the condensed phase, the chemical structure of PS/GO2.0 and PS/FGO–AHP5.0 residual char was characterized by FTIR (Figure 11). For PS/GO2.0, the peaks at 1550 and 1650 cm^−1^ are assigned to the vibration of benzene rings [53]. The absorption peak at 1187 cm^−1^ for C-O bonds indicates the oxidation of graphene under combustion [54]. The characteristic peaks at 718 and 610 cm^−1^ are due to the C-H out-of-plane bending vibration [32]. In the spectrum of PS/FGO–AHP5.0, the new characteristic peak at 1165 cm^−1^ corresponds to the PO_3_^2−^/PO_4_^3−^ anion or the characteristic of P-O-Ar, demonstrating the formation of a phosphocarbonaceous structure in the char [55]. The stretch vibration of P-O-P and P-O-Ph can be reflected at 998 cm^−1^ and 684 cm^−1^, respectively, in polyphosphate species. The strong peak at 483 cm^−1^ is ascribed to the degradation product of AHP (Al-O bond stretch modes), suggesting that the aluminum phosphate can further facilitate the charring cross-linking reaction, providing an effective barrier against mass transfer and heat diffusion [56]. These results reveal the presence of phosphorus-containing compounds and aluminum hypophosphite in the condensed phase, which contribute to forming a more compact char layer, as confirmed by both SEM and Raman spectroscopy results. The resulting char residue can serve as an effective barrier to restrain heat and mass transfer and weaken exothermic reactions, optimizing the fire safety performance of PS nanocomposites in condensed and gas phases.

## 4. Conclusions

In conclusion, an organic–inorganic nanohybrid material, FGO–AHP, was prepared by combining phosphorus-containing molecules functionalized GO with aluminum hypophosphite. Microstructural characterization confirms the successful preparation of FGO–AHP nanohybrids. The prepared FGO–AHP nanohybrids were melt blended with PS to obtain PS/FGO–AHP nanocomposites. SEM observation shows a uniform dispersion of FGO–AHP in PS nanocomposites, which can be attributed to the strong interaction between the nanohybrids and the PS matrix. The TGA results suggest that the well-designed FGO–AHP nanofillers significantly improve the high-temperature stability of UPR, leading to a much-decreased MMLR and a notably enhanced char yield in PS/FGO–AHP nanocomposites. Moreover, microcalorimetry and smoke density tests show that the incorporation of FGO–AHP into PS significantly reduces heat release and toxic smoke emission, as the PHRR and D_smax_ values of PS/FGO–AHP5.0 are reduced by 39.9% and 50.9%, respectively. TG–FTIR and char residue analysis results confirm the optimized fire safety performance in PS/FGO–AHP nanocomposites. These improvements are attributed to the catalytic char formation in the condensed phase, the physical barrier effect, and the quenching effect in the vapor phase. The proposed strategy for hybridizing FGO and AHP harnesses the advantages of graphene nanosheets and AHP for enhanced fire safety protection, establishing a solid foundation for the development of high-performance flame retardants.

## Figures and Tables

**Table 1:** TG data of GO, FGO, and FGO–AHP in N_2_ condition.

Sample	T_0.1_ (°C)	T_0.2_ (°C)	Char Residue (800 °C, wt%)
GO	117.4	211.7	48.2
FGO	189.3	274.5	55.1
FGO–AHP	295.6	334.3	73.6

**Table 2:** TGA data of PS and PS nanocomposites in nitrogen and air conditions.

Samples	T_0.1_ (°C) (N_2_/Air)	T_max_ (°C) (N_2_/Air)	MMLR (%/°C) (N_2_/Air)	Residues at 700 °C (wt%) (N_2_/Air)
Error	±1	±1	±0.01	±0.01
PS	395/335	420/378	2.76/2.75	0.53/0.46
PS/GO2.0	397/322	420/384	2.60/2.00	0.61/1.04
PS/FGO2.0	401/332	416/390	2.49/1.98	0.89/1.34
PS/FGO–AHP2.0	378/330	420/379	2.45/1.82	1.19/1.80
PS/FGO–AHP3.0	382/325	420/384	2.42/1.46	1.83/2.49
PS/FGO–AHP4.0	386/323	419/381	2.40/1.38	3.24/3.32
PS/FGO–AHP5.0	388/321	418/383	2.34/1.29	4.66/4.73

**Table 3:** MCC data of PS and PS nanocomposites.

Samples	PHRR (W/g)	HRC (J/g·K)	THR (kJ/g)
Error	±1	±1	±0.1
PS	1063	1051	45.6
PS/GO2.0	964	963	42.5
PS/FGO2.0	925	921	41.4
PS/FGO–AHP2.0	774	764	39.6
PS/FGO–AHP3.0	708	702	37.9
PS/FGO–AHP4.0	664	656	35.8
PS/FGO–AHP5. 0	639	627	34.9

**Table 4:** Comparison of the flame retardancy of various fillers in PS matrix.

Matrix	Filler (wt%)	Reduction in PHRR (%)	Reduction in THR (%)	Reference
PS	TGO (5%)	23.9%	20.0%	[3]
PS	MP-EG (20%)	35.3%	21.8%	[47]
PS	hBN-SBC (20%)	29.0%	14.1%	[48]
PS	SiAPP/aMWCNT (20%)	29.6%	25.2%	[49]
PS	Fc-GNS (2%)	27.0%	12.0%	[50]
PS	FGO–AHP (2%)	27.2%	13.2%	Present work
PS	FGO–AHP (3%)	33.4%	16.9%	Present work
PS	FGO–AHP (4%)	37.5%	21.5%	Present work
PS	FGO–AHP (5%)	39.9%	23.5%	Present work

## Data Availability

Data are contained within the article and Supplementary Materials.

## Supplementary Materials

The following supporting information can be downloaded at: https://www.mdpi.com/article/10.3390/polym16213083/s1, Table S1: Tensile testing data of PS and PS nanocomposites.

## References

[1] Zhang F., Yang K., Liu G., Chen Y., Wang M., Li S., Li R. (2022). Recent advances on graphene: Synthesis, properties and applications. Compos. Part A Appl. Sci. Manuf..

[2] Govindaraj P., Sokolova A., Salim N., Juodkazis S., Fuss F.K., Fox B., Hameed N. (2021). Distribution states of graphene in polymer nanocomposites: A review. Compos. Part B Eng..

[3] Han Y., Wang T., Gao X., Li T., Zhang Q. (2016). Preparation of thermally reduced graphene oxide and the influence of its reduction temperature on the thermal, mechanical, flame retardant performances of PS nanocomposites. Compos. Part A Appl. Sci. Manuf..

[4] Kalali E.N., Guo W., Wang X., Xing W., Song L., Hu Y. (2020). Effect of metal-based nanoparticles decorated graphene hybrids on flammability of epoxy nanocomposites. Compos. Part A Appl. Sci. Manuf..

[5] Gnanasekar P., Chen H., Tratnik N., Feng M., Yan N. (2021). Enhancing performance of phosphorus containing vanillin-based epoxy resins by P–N non-covalently functionalized graphene oxide nanofillers. Compos. Part B Eng..

[6] Yu W., Sisi L., Haiyan Y., Jie L. (2020). Progress in the functional modification of graphene/graphene oxide: A review. RSC Adv..

[7] Zhang Z., Yuan L., Guan Q., Liang G., Gu A. (2017). Synergistically building flame retarding thermosetting composites with high toughness and thermal stability through unique phosphorus and silicone hybridized graphene oxide. Compos. Part A Appl. Sci. Manuf..

[8] Sang B., Li Z.-W., Li X.-H., Yu L.-G., Zhang Z.-J. (2016). Graphene-based flame retardants: A review. J. Mater. Sci..

[9] Liao S.-H., Liu P.-L., Hsiao M.-C., Teng C.-C., Wang C.-A., Ger M.-D., Chiang C.-L. (2012). One-step reduction and functionalization of graphene oxide with phosphorus-based compound to produce flame-retardant epoxy nanocomposite. Ind. Eng. Chem. Res..

[10] Hou Y., Hu W., Liu L., Gui Z., Hu Y. (2018). In-situ synthesized CNTs/Bi_2_Se_3_ nanocomposites by a facile wet chemical method and its application for enhancing fire safety of epoxy resin. Compos. Sci. Technol..

[11] Feng Y., Li X., Zhao X., Ye Y., Zhou X., Liu H., Liu C., Xie X. (2018). Synergetic improvement in thermal conductivity and flame retardancy of epoxy/silver nanowires composites by incorporating “branch-like” flame-retardant functionalized graphene. ACS Appl. Mater. Interfaces.

[12] Chen X., Yun Y., Fan A., Yuan B., Shang S., He S. (2019). The assembly nanohybrid of graphene with lamellar zirconium phenylphosphonate for improving flame retardancy and mechanical properties of polypropylene. Polym. Compos..

[13] Zhu M., Liu L., Wang Z. (2020). Iron-phosphorus-nitrogen functionalized reduced graphene oxide for epoxy resin with reduced fire hazards and improved impact toughness. Compos. Part B Eng..

[14] Hu W., Yu B., Jiang S.-D., Song L., Hu Y., Wang B. (2015). Hyper-branched polymer grafting graphene oxide as an effective flame retardant and smoke suppressant for polystyrene. J. Hazard. Mater..

[15] Hou Y., Liu L., Qiu S., Zhou X., Gui Z., Hu Y. (2018). DOPO-modified two-dimensional Co-based metal–organic framework: Preparation and application for enhancing fire safety of poly (lactic acid). ACS Appl. Mater. Interfaces.

[16] Shi X., Peng X., Zhu J., Lin G., Kuang T. (2018). Synthesis of DOPO-HQ-functionalized graphene oxide as a novel and efficient flame retardant and its application on polylactic acid: Thermal property, flame retardancy, and mechanical performance. J. Colloid Interface Sci..

[17] Shi Y., Yu B., Zheng Y., Yang J., Duan Z., Hu Y. (2018). Design of reduced graphene oxide decorated with DOPO-phosphanomidate for enhanced fire safety of epoxy resin. J. Colloid Interface Sci..

[18] Mihis A.G., Cotet L.C., Cadar C., Pop L.C., Todea M., Rusu M.M., Vulpoi A., Székely I., Sălăgean C.A., Magyari K. (2023). Structural and flame retardancy properties of GO-DOPO-HAK composite. J. Mater. Sci..

[19] Liu M., Xie Z., Ye H., Li W., Shi W., Liu Y., Zhang Y. (2021). Waste polystyrene foam–Chitosan composite materials as high-efficient scavenger for the anionic dyes. Colloids Surf. A.

[20] Lu J., Wang D., Jiang P., Zhang S., Chen Z., Bourbigot S., Fontaine G., Wei M. (2021). Design of fire resistant, sound-absorbing and thermal-insulated expandable polystyrene based lightweight particleboard composites. Constr. Build. Mater..

[21] Benchouia H.E., Boussehel H., Guerira B., Sedira L., Tedeschi C., Becha H.E., Cucchi M. (2024). An experimental evaluation of a hybrid bio-composite based on date palm petiole fibers, expanded polystyrene waste, and gypsum plaster as a sustainable insulating building material. Constr. Build. Mater..

[22] Zhu Y., Shi Y., Huang Z.-Q., Duan L., Tai Q., Hu Y. (2017). Novel graphite-like carbon nitride/organic aluminum diethylhypophosphites nanohybrid: Preparation and enhancement on thermal stability and flame retardancy of polystyrene. Compos. Part A Appl. Sci. Manuf..

[23] Hou B., Song X., Song K., Geng Z., Pan Y.-T., Song P., Yang R. (2024). Synchronous preparation and modification of LDH hollow polyhedra by polydopamine: Synthesis and application. J. Colloid Interface Sci..

[24] Ye T.-P., Liao S.-F., Zhang Y., Chen M.-J., Xiao Y., Liu X.-Y., Liu Z.-G., Wang D.-Y. (2019). Cu (0) and Cu (II) decorated graphene hybrid on improving fireproof efficiency of intumescent flame-retardant epoxy resins. Compos. Part B Eng..

[25] Xu B., Liu Y., Wei S., Zhao S., Qian L., Chen Y., Shan H., Zhang Q. (2022). A phosphorous-based bi-functional flame retardant based on phosphaphenanthrene and aluminum hypophosphite for an epoxy thermoset. Int. J. Mol. Sci..

[26] Tawiah B., Yu B., Fei B. (2018). Advances in flame retardant poly (lactic acid). Polymers.

[27] Li J., Qin Z., Zhai C., Yang R. (2024). Study on the synergistic flame-retardancy of phenyl/vinyl siliconesquioxane and aluminum diethyl phosphinate on polyethylene terephthalate. Polym. Degrad. Stab..

[28] Lou Y., Ma H., Jia Y., Sun S., Wang M., Xu J. (2024). One-pot synthesis of aluminum hypophosphite encapsulation in expanded halloysite lumen for reducing fire hazard and environmental pollution. Polym. Degrad. Stab..

[29] Ge H., Tang G., Hu W.-Z., Wang B.-B., Pan Y., Song L., Hu Y. (2015). Aluminum hypophosphite microencapsulated to improve its safety and application to flame retardant polyamide 6. J. Hazard. Mater..

[30] Bansal S., Prakash K., Sharma K., Sardana N., Kumar S., Gupta N., Singh A.K. (2020). A highly efficient bilayer graphene/ZnO/silicon nanowire based heterojunction photodetector with broadband spectral response. Nanotechnology.

[31] Bansal S., Das A., Prakash K., Sharma K., Khanal G.M., Sardana N., Kumar S., Gupta N., Singh A.K. (2022). Bilayer graphene/HgCdTe heterojunction based novel GBn infrared detectors. Micro Nanostruct..

[32] Dai K., Sun S., Xu W., Song Y., Deng Z., Qian X. (2018). Covalently-functionalized graphene oxide via introduction of bifunctional phosphorus-containing molecules as an effective flame retardant for polystyrene. RSC Adv..

[33] Feng Y., Hu J., Xue Y., He C., Zhou X., Xie X., Ye Y., Mai Y.-W. (2017). Simultaneous improvement in the flame resistance and thermal conductivity of epoxy/Al_2_O_3_ composites by incorporating polymeric flame retardant-functionalized graphene. J. Mater. Chem. A.

[34] Ji P., Cui Y., Liu D., Zhang T., Lv J. (2021). The Bi-DOPO derivative functionalized graphene oxide: Preparation and its flame-retardation on epoxy resin. Polym. Adv. Technol..

[35] Lin Y., Jiang S., Hu Y., Chen G., Shi X., Peng X. (2018). Hybrids of aluminum hypophosphite and ammonium polyphosphate: Highly effective flame retardant system for unsaturated polyester resin. Polym. Compos..

[36] Xu W., Wang X., Wu Y., Li W., Chen C. (2019). Functionalized graphene with Co-ZIF adsorbed borate ions as an effective flame retardant and smoke suppression agent for epoxy resin. J. Hazard. Mater..

[37] Zhang Q., Li Z., Li X., Yu L., Zhang Z., Wu Z. (2019). Zinc ferrite nanoparticle decorated boron nitride nanosheet: Preparation, magnetic field arrangement, and flame retardancy. Chem. Eng. J..

[38] Wang H., Wang Z., Shi Y., Fu L., Liu M., Feng Y., Yu B., Gao J., Yang F. (2022). Interface assembly of hypophosphite/ultrathin MXene nanosheets towards fire safe polylactic acid composites. Compos. Commun..

[39] Peyravi A., Ahmadijokani F., Arjmand M., Hashisho Z. (2022). Graphene oxide enhances thermal stability and microwave absorption/regeneration of a porous polymer. J. Hazard. Mater..

[40] Imran M., Raza M., Noor H., Faraz S.M., Raza A., Farooq U., Khan M.E., Ali S.K., Bakather O.Y., Ali W. (2024). Insight into mechanism of excellent visible-light photocatalytic activity of CuO/MgO/ZnO nanocomposite for advanced solution of environmental remediation. Chemosphere.

[41] Wang J., Jin X., Li C., Wang W., Wu H., Guo S. (2019). Graphene and graphene derivatives toughening polymers: Toward high toughness and strength. Chem. Eng. J..

[42] He W., Song P., Yu B., Fang Z., Wang H. (2020). Flame retardant polymeric nanocomposites through the combination of nanomaterials and conventional flame retardants. Prog. Mater. Sci..

[43] Noor H., Zafar A., Raza A., Baqi A., Farooq U., Khan M.E., Ali W., Ali S.K., Bashiri A.H., Zakri W. (2024). Advancement in optical and dielectric properties of unsaturated polyester resin/zinc oxide nanocomposite: Synthesis to application in electronics. J. Mater. Sci. Mater. Electron..

[44] Wiacek M., Wesolek D., Rojewski S., Bujnowicz K., Schab-Balcerzak E. (2014). Halogeno-modified polystyrene: Monomer reactivity ratios, thermal behaviour and flammability. Polym. Int..

[45] Hou Y., Hu W., Hu Y. (2017). Preparation of layered organic-inorganic aluminum phosphonate for enhancing fire safety of polystyrene. Mater. Chem. Phys..

[46] Zhu Z.-M., Rao W.-H., Kang A.-H., Liao W., Wang Y.-Z. (2018). Highly effective flame retarded polystyrene by synergistic effects between expandable graphite and aluminum hypophosphite. Polym. Degrad. Stab..

[47] Wang G., Bai S. (2017). Synergistic effect of expandable graphite and melamine phosphate on flame-retardant polystyrene. J. Appl. Polym. Sci..

[48] Amirabadi S., Kheradmandkeysomi M., Zandieh A., Serles P., Tanguy N., Filleter T., Sain M., Park C.B. (2024). Highly tough and flame retardant polystyrene composites by elastomeric nanofibers and hexagonal boron nitride. J. Mater. Sci. Technol..

[49] Huang R., Gao C., Shi Y., Fu L., Feng Y., Shui W. (2022). Synergistic function between phosphorus-containing flame retardant and multi-walled carbon nanotubes towards fire safe polystyrene composites with enhanced electromagnetic interference shielding. Int. J. Mol. Sci..

[50] Zhou K., Gui Z., Hu Y. (2016). The influence of graphene based smoke suppression agents on reduced fire hazards of polystyrene composites. Compos. Part A Appl. Sci. Manuf..

[51] Hu G., Zhang X., Bu M., Lei C. (2022). Toughening and strengthening epoxy resins with a new bi-DOPO biphenyl reactive flame retardant. Eur. Polym. J..

[52] Liu M., Gong Z., Wang G., Liu X., Hou Y., Tang G. (2024). Melamine resin coordinated cobalt@piperazine pyrophosphate microcapsule: An innovative strategy for imparting long-lasting fire safety to rigid polyurethane foams. Polym. Degrad. Stab..

[53] Xia Z., Kiratitanavit W., Facendola P., Thota S., Yu S., Kumar J., Mosurkal R., Nagarajan R. (2018). Fire resistant polyphenols based on chemical modification of bio-derived tannic acid. Polym. Degrad. Stab..

[54] Yuan B., Hu Y., Chen X., Shi Y., Niu Y., Zhang Y., He S., Dai H. (2017). Dual modification of graphene by polymeric flame retardant and Ni(OH)_2_ nanosheets for improving flame retardancy of polypropylene. Compos. Part A Appl. Sci. Manuf..

[55] Wang H., Li S., Zhu Z., Yin X., Wang L., Weng Y., Wang X. (2021). A novel DOPO-based flame retardant containing benzimidazolone structure with high charring ability towards low flammability and smoke epoxy resins. Polym. Degrad. Stab..

[56] Tang G., Wang X., Xing W., Zhang P., Wang B., Hong N., Yang W., Hu Y., Song L. (2012). Thermal degradation and flame retardance of biobased polylactide composites based on aluminum hypophosphite. Ind. Eng. Chem. Res..

