# Supplementary material for: Fabrication of Functionalized Graphene Oxide–Aluminum Hypophosphite Nanohybrids for Enhanced Fire Safety Performance in Polystyrene
Source: Polymers (Basel). 2024 Oct 31;16(21):3083. doi: 10.3390/polym16213083 (PMC11548397; doi:10.3390/polym16213083)
Supplement: Supplementary file 1 [file polymers-16-03083-s001.zip › polymers-3261951-supplementary.pdf]

Supporting Information

Fabrication of Functionalized Graphene Oxide–Aluminum Hypophosphite Nanohybrids for Enhanced Fire Safety Performance in Polystyrene

Zhenzhen Deng [1], Tao Tang [1,2], Junjie Huo [1], Hui He [1] and Kang Dai [1,*]

[1] School of Environmental Science and Engineering, Guangdong University of Technology,
Guangzhou 510006, China; dzz_dengzhenzhen@163.com (Z.D.);
18183375687@163.com (T.T.); 2112207192@mail2.gdut.edu.cn (J.H.);
hehui2737@163.com (H.H.)
[2] JIAHUA Special Cement Co., Ltd., Leshan 614003, China
* Correspondence: daikang@mail.ustc.edu.cn

Mechanical properties of PS and PS nanocomposites

The mechanical performance of PS and PS nanocomposites was evaluated by tensile testing. As presented in Table S1, incorporating nanofillers into PS improves the tensile properties of PS nanocomposites. Different from the limited tensile strength enhancement in PS/GO2.0, the tensile strength of PS/FGO2.0 and PS/FGO-AHP2.0 are increased by 31.1% and 24.0%, respectively, as compared to that of neat PS. The surface functionalization of GO effectively ameliorates the dispersion of FGO and FGO-AHP in the PS matrix, reinforcing the interfacial reaction between nanofillers and PS molecular chains [1]. The highest tensile strength and elastic modulus can be seen in PS/FGO3.0, and further increasing the amount of FGO-AHP nanohybrids in PS nanocomposites shows weakened reinforcing efficiency in tensile properties. However, the tensile strength and elastic modulus of PS/FGO-AHP5.0 reach 13.11 and 1382 MPa, respectively. The enhanced mechanical performance in PS/FGO-AHP composites confirms the favorable interaction between the PS matrix and FGO-AHP nanohybrids, fitting well with the fractured surface SEM results.

Table S1. Tensile testing data of PS and PS nanocomposites.

Sample	Tensile strength (MPa)	Elastic modulus (MPa)
PS	9.29±0.24	1012±46
PS/GO2.0	10.59±0.23	1203± 35
PS/FGO2.0	12.18±0.19	1358±32
PS/FGO-AHP2.0	11.52 ±0.22	1306±27
PS/FGO-AHP3.0	13.66 ±0.18	1455±31
PS/FGO-AHP4.0	13.58 ±0.20	1447±33
PS/FGO-AHP5.0	13.11 ±0.17	1382±29

1. Wang, J.; Jin, X.; Li, C.; Wang, W.; Wu, H.; Guo, S. Graphene and graphene derivatives toughening polymers: Toward high toughness and strength. *Chem. Eng. J.* **2019**, 370, 831-854.
